# Supplementary material for: Development of Attenuated Viruses for Effective Protection against Pepper Veinal Mottle Virus in Tomato Crops
Source: Viruses. 2024 Apr 26;16(5):687. doi: 10.3390/v16050687 (PMC11125906; doi:10.3390/v16050687)
Supplement: Supplementary file 1 [file viruses-16-00687-s001.zip › Fig S1_PVMV accumulation in Nb.pdf]

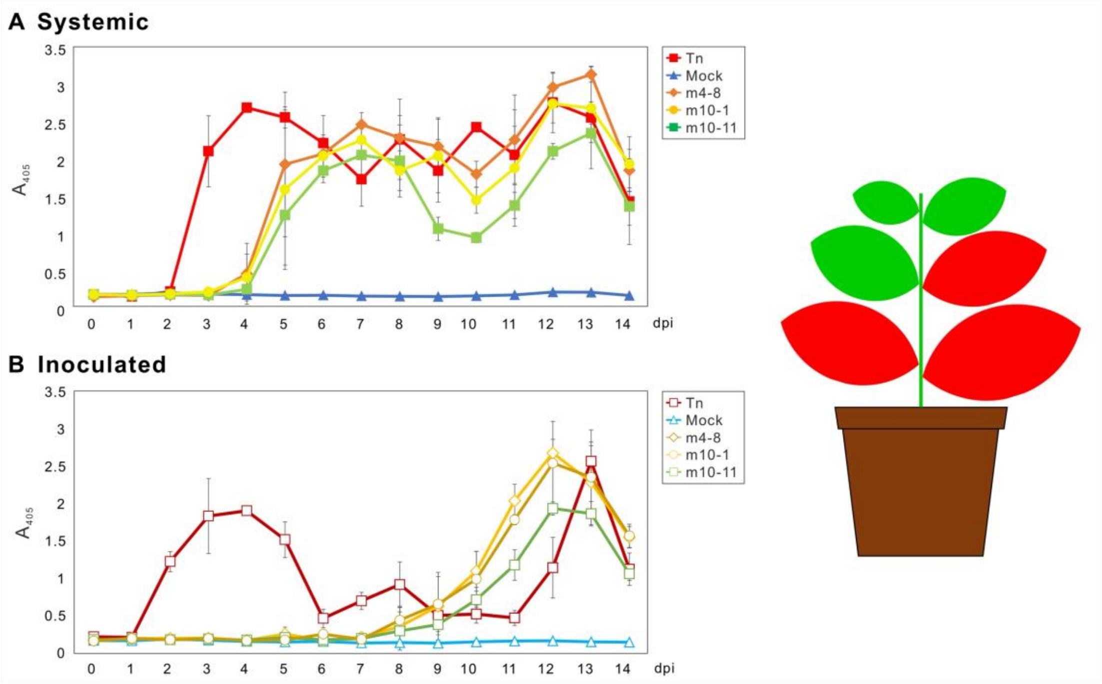

Figure S1. Accumulation of wild-type (WT) pepper veinal mottle virus (PVMV) Tn and its attenuated mutants, m4-8, m10-1 and m10-11, in *Nicotiana benthamiana* plants. Indirect enzyme-linked immunosorbent assay (ELISA) with the antiserum against the coat protein of PVMV was conducted to assess virus accumulation. Crude leaf saps of virus-inoculated plants 0-14 days post-inoculation (dpi) at a 1/100 dilution were used for virus detection. Non-inoculated systemic leaves **(A)** and inoculated leaves **(B)** were separately tested. The positions of systemic leaves (green) and inoculated leaves (red) are indicated on the right.
