# Supplementary material for: Development of Attenuated Viruses for Effective Protection against Pepper Veinal Mottle Virus in Tomato Crops
Source: Viruses. 2024 Apr 26;16(5):687. doi: 10.3390/v16050687 (PMC11125906; doi:10.3390/v16050687)
Supplement: Supplementary file 1 [file viruses-16-00687-s001.zip › Fig S2_Protection and DSI in Nb.pdf]

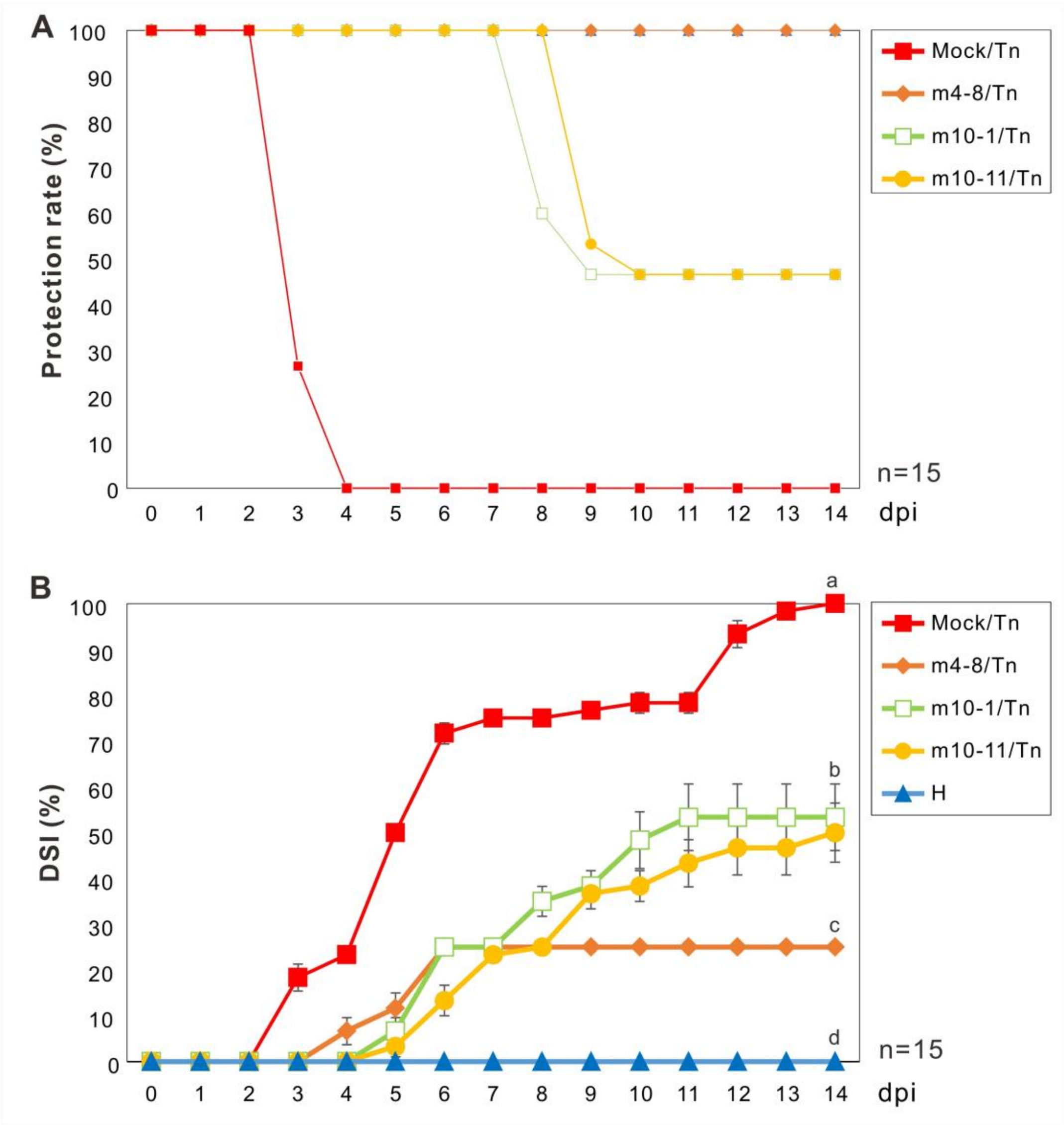

Figure S2. Evaluation of protective effectiveness of the pepper veinal mottle virus (PVMV) mutants m4-8, m10-1, and m10-11 in *Nicotiana benthamiana* plants. Test plants were challenged with the wild-type PVMV Tn 4 days after inoculation with the protectors. Mock-inoculated plants with or without challenge served as controls. A total of 15 plants were used per treatment. **(A)** The protection rate of each attenuated mutant. **(B)** Disease severity index (DSI) was estimated with the formula [sum (type frequency × score of rating class)] / [(total number of plants) × (maximal disease index)] × 100% [35]. Symptom development was scored 14 days after inoculation of attenuated mutants. Scores for disease severity rating were given for each plant, and 15 plants per treatment were assayed to obtain an average value. Healthy plants (H) have an index scale of 0. Significant differences among treatments were analyzed by the Mann-Whitney *U* test, $p < 0.05$ [37].
