# Supplementary material for: Development of Attenuated Viruses for Effective Protection against Pepper Veinal Mottle Virus in Tomato Crops
Source: Viruses. 2024 Apr 26;16(5):687. doi: 10.3390/v16050687 (PMC11125906; doi:10.3390/v16050687)
Supplement: Supplementary file 1 [file viruses-16-00687-s001.zip › Fig S3_Protection against Xs-1 in Nb.pdf]

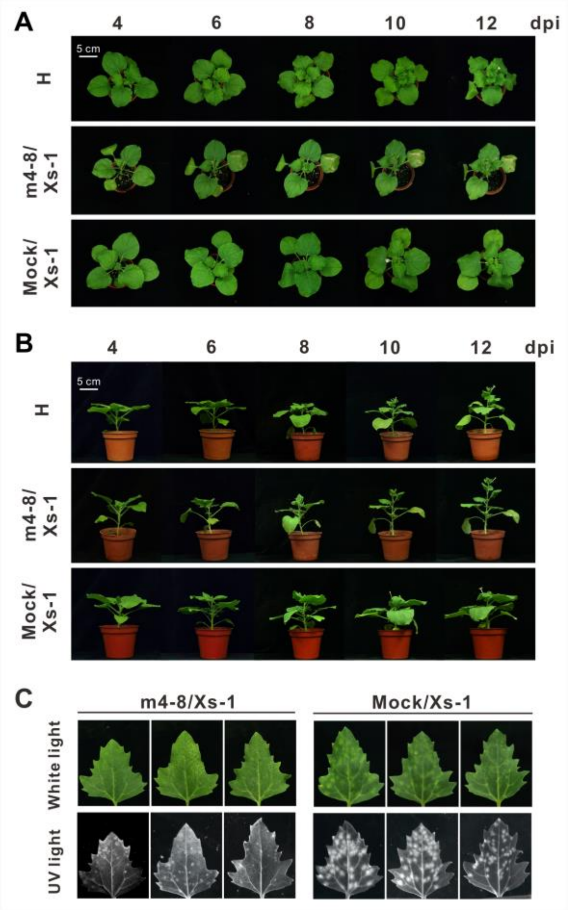


**Figure S3. Evaluation of the protective effect of the pepper veinal mottle virus (PVMV) attenuated mutant m4-8 against the different PVMV isolate Xs-1 in *Nicotiana benthamiana* plants. (A and B)** Observation on symptom development and growth potential of test *N. benthamiana* plants. Plants 4 days after inoculation with m4-8 were challenged with Xs-1. Symptom development was recorded daily for 12 days. Ten plants were used for each treatment. **(C)** Xs-1 infection was assessed by bioassay on *Chenopodium quinoa* leaves. Local lesions were observed under white light (upper panel) and UV light (365 nm) (lower panel) 10 days post-inoculation.
