# Supplementary material for: Development of Attenuated Viruses for Effective Protection against Pepper Veinal Mottle Virus in Tomato Crops
Source: Viruses. 2024 Apr 26;16(5):687. doi: 10.3390/v16050687 (PMC11125906; doi:10.3390/v16050687)
Supplement: Supplementary file 1 [file viruses-16-00687-s001.zip › Fig S4_Protection days.pdf]

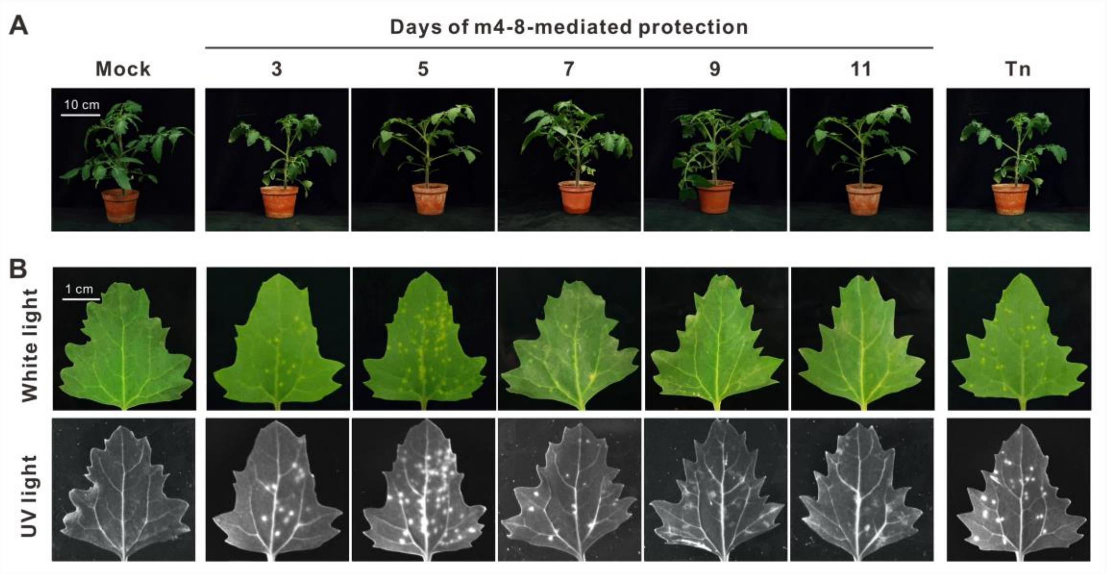

Figure S4. Protection time assay for cross-protection of pepper veinal mottle virus (PVMV) mutant m4-8 against the wild-type PVMV Tn in tomato plants. **(A)** Tomato plants treated with m4-8 were challenged with PVMV Tn at 3, 5, 7, 9, and 11 days post-inoculation (dpi). Symptom development was observed 14 days after the challenge with PVMV Tn. **(B)** Bioassay was conducted on *Chenopodium quinoa* leaves to assess PVMV Tn infection. Local lesions were observed under white light (upper panel) and UV light illumination (365 nm) (lower panel) at 10 dpi. Mock inoculation was used as the negative control.
