# Supplementary material for: Development of Attenuated Viruses for Effective Protection against Pepper Veinal Mottle Virus in Tomato Crops
Source: Viruses. 2024 Apr 26;16(5):687. doi: 10.3390/v16050687 (PMC11125906; doi:10.3390/v16050687)
Supplement: Supplementary file 1 [file viruses-16-00687-s001.zip › Fig S5_HC-Pro alignment.pdf]

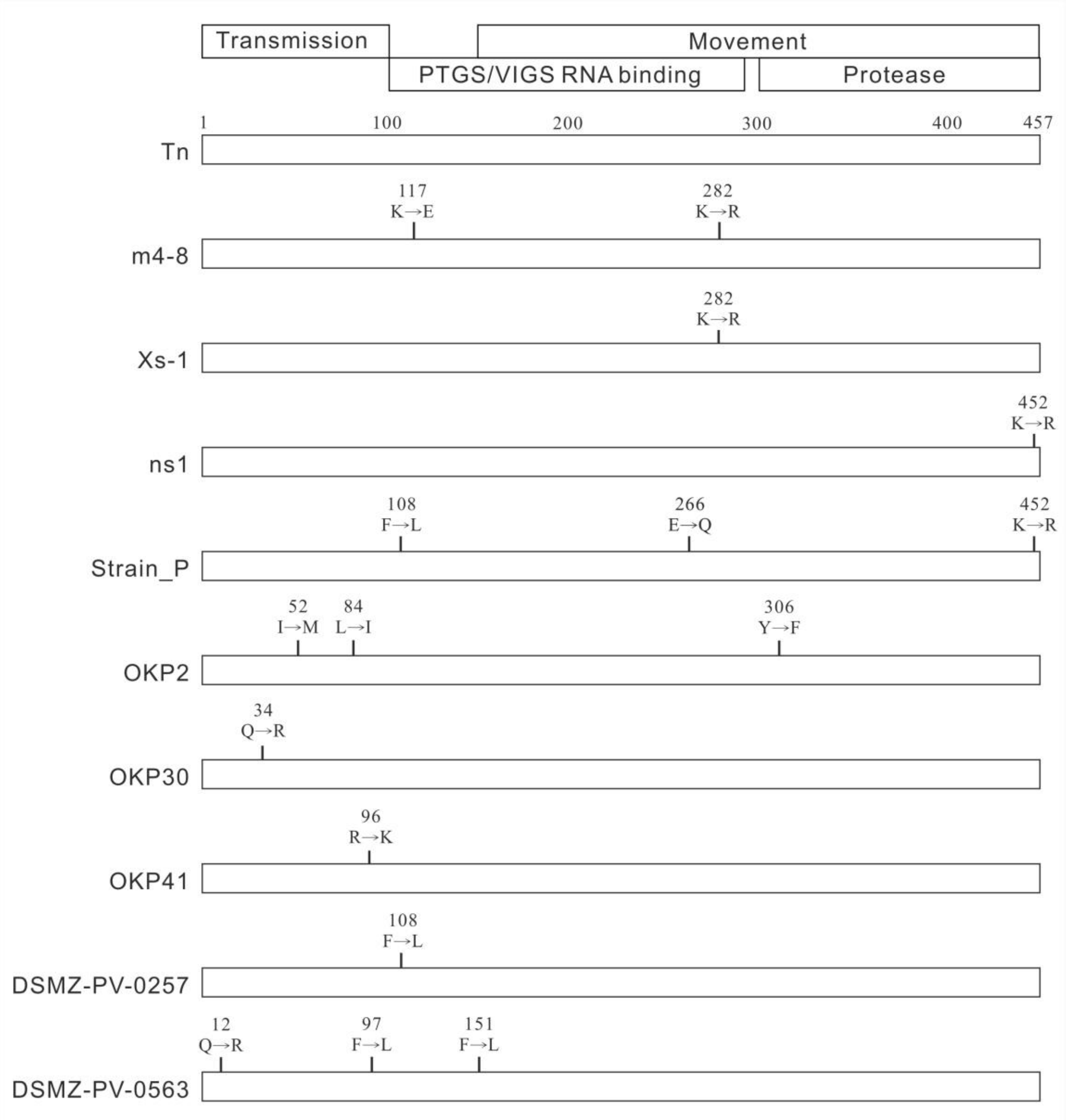


**Figure S5. Amino acid differences within the HC-Pro of different pepper veinal mottle virus (PVMV) isolates.** The HC-Pro of PVMV Tn was used as a comparison standard. The letters on the left indicate the amino acid residues of PVMV Tn. The letters on the right indicate different amino acid residues.
