# Supplementary material for: Development of Attenuated Viruses for Effective Protection against Pepper Veinal Mottle Virus in Tomato Crops
Source: Viruses. 2024 Apr 26;16(5):687. doi: 10.3390/v16050687 (PMC11125906; doi:10.3390/v16050687)
Supplement: Supplementary file 1 [file viruses-16-00687-s001.zip › Fig S6_Fruit yield.pdf]

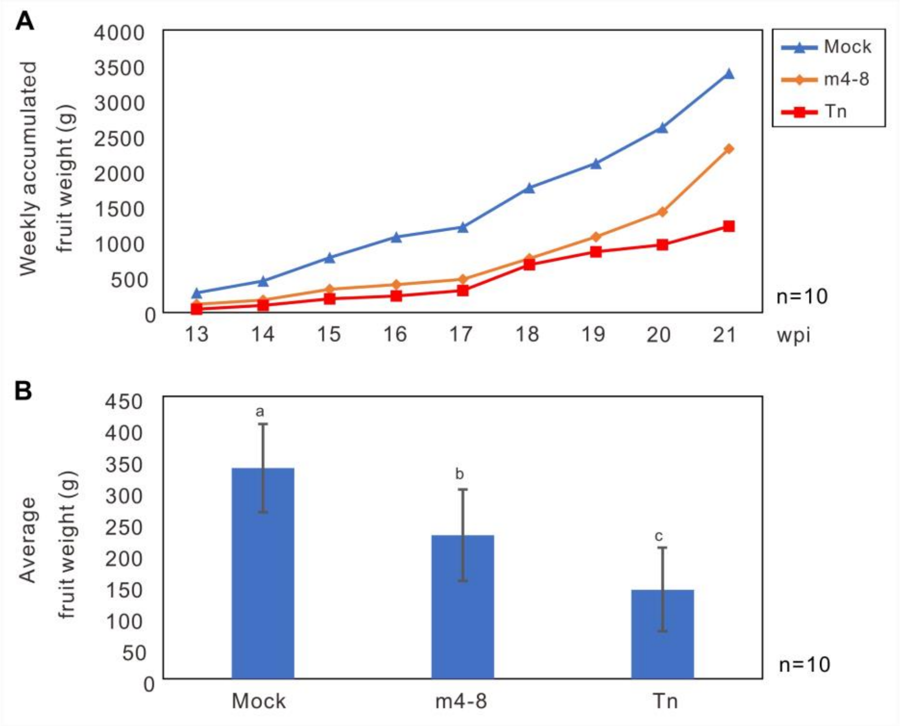

Figure S6. Fruit yield evaluation of tomato plants protected with the pepper veinal mottle virus (PVMV) mutant m4-8 under greenhouse conditions. (A) The weekly accumulation of tomato fruit yield (n=10) was recorded at 13-21 weeks post-inoculation (wpi). **(B)** Average total tomato fruit yield per tomato plant (n=10). Different letters (a-c) above the bars denote significant differences (Mann-Whitney *U* test, $p < 0.05$) [37]. Mock inoculation was used for comparison.
