# Supplementary material for: Development of Attenuated Viruses for Effective Protection against Pepper Veinal Mottle Virus in Tomato Crops
Source: Viruses. 2024 Apr 26;16(5):687. doi: 10.3390/v16050687 (PMC11125906; doi:10.3390/v16050687)
Supplement: Supplementary file 1 [file viruses-16-00687-s001.zip › Table S1_Primers-1.pdf]

Table S1. Nucleotide sequences of the primers used in this study.

Primer name	Direction[a]	5' → 3'
PVMV-TW-1-Stu	F	GCAAGTACAGGCCTAAAAAATTAAACATCTCATACAAACAAATACGAAA
PVMV-3031c	R	CCGCCTGTACATGACTTTCTTG
PVMV-2361	F	CACTAACAACTGGGTACCACATCCTCAAAGCTAACAC
PVMV-5681c	R	GCCACACCAGCACCAATCAA
PVMV-5561	F	CGGCGCTATGGAAACTGTCA
PolyT Not	R	CGTTTTTTTTGCGGCCGCTTTTTTTTTTTTTTTT

[a] F and R represent the primers are designed in forward and reverse directions, respectively.
