# Supplementary material for: Development of Attenuated Viruses for Effective Protection against Pepper Veinal Mottle Virus in Tomato Crops
Source: Viruses. 2024 Apr 26;16(5):687. doi: 10.3390/v16050687 (PMC11125906; doi:10.3390/v16050687)
Supplement: Supplementary file 1 [file viruses-16-00687-s001.zip › Table S2_Genome comparison.pdf]

Table S2. Whole-genome comparison of pepper veinal mottle virus (PVMV) isolate Tn with other PVMV isolates.

Virus isolate	Accession number	Host	Location	nt (%)	aa (%)
Tn	OR355467	*Solanum lycopersicum*	Taiwan	100.0	100.0
Xs-1	OR355466	*Capsicum annuum*	Taiwan	97.5	97.7
ns1	FJ617225	*S. nigrum*	Taiwan	97.8	98.0
HNu	MN082715	*C. chinense*	China	98.0	98.2
OKP2	LC438540	*C. annuum*	Japan	97.8	97.9
OKP3	LC438541	*C. annuum*	Japan	98.0	98.0
OKC25	LC438542	*C. annuum*	Japan	97.8	97.9
OKP30	LC438543	*C. annuum*	Japan	97.8	97.8
OKP39	LC438544	*C. annuum*	Japan	97.8	97.9
OKP41	LC438545	*C. frutescens*	Japan	97.8	97.8
P	NC_011918	*C. frutescens*	Ghana	98.1	97.1
DSMZ PV-0257	MZ405642	*C. frutescens*	Ghana	98.3	98.1
DSMZ PV-0563	OP722584	*S. lycopersicum*	Nigeria	93.7	95.4
DSMZ PV-0551	OQ102061	*Capsicum* spp.	Nigeria	93.1	95.1
Camb 2	OK558746	*Capsicum* spp.	Senegal	83.7	89.8
Malika-1	OK558747	*Capsicum* spp.	Senegal	93.2	95.2
